# Perfluorooctanoate in Aqueous Urea Solutions: Micelle Formation, Structure, and Microenvironment

**DOI:** 10.3390/ijms20225761

**Published:** 2019-11-16

**Authors:** Samhitha Kancharla, Emmanuel Canales, Paschalis Alexandridis

**Affiliations:** Department of Chemical and Biological Engineering, University at Buffalo, The State University of New York (SUNY), Buffalo, NY 14260, USA; skanchar@buffalo.edu (S.K.); ecanales@buffalo.edu (E.C.)

**Keywords:** perfluoroalkyl substances (PFAS), water, surfactant, urea, denaturation

## Abstract

Fluorinated surfactants are used in a wide range of applications that involve aqueous solvents incorporating various additives. The presence of organic compounds such as urea is expected to affect the self-assembly of fluorinated surfactants, however, very little is known about this. We investigated the effect of urea on the micellization in water of the common fluorinated surfactant ammonium perfluorooctanoate (APFO), and on the structure and microenvironment of the micelles that APFO forms. Addition of urea to aqueous APFO solution decreased the critical micellization concentration (CMC) and increased the counterion dissociation. The observed increase in surface area per APFO headgroup and decrease in packing density at the micelle surface suggest the localization of urea at the micelle surface in a manner that reduces headgroup repulsions. Micropolarity data further support this picture. The results presented here indicate that significant differences exist between urea effects on fluorinated surfactant and on hydrocarbon surfactant micellization in aqueous solution. For example, the CMC of sodium dodecyl sulfate (SDS) increased with urea addition, while the increase in surface area per headgroup and packing density of SDS with urea addition are much lower than those observed for APFO. This study informs fluorinated surfactant fate and transport in the environment, and also applications involving aqueous media in which urea or similar additives are present.

## 1. Introduction

Urea is a commonly used protein denaturant in aqueous solutions [1,2]. Urea also assists the dissolution in aqueous media of difficult-to-dissolve polymers, such as cellulose [3,4]. The mechanism of urea action and the structure in aqueous urea solutions remain contentious. Some studies have indicated that urea acts as a water structure breaker [2,5,6,7], other studies suggest that urea enhances the water structure and acts as a weak water structure maker [8,9], and few studies reported that urea has no effect on the water structure [10,11,12,13,14]. The controversy on the effect of urea on the water structure network arises partly because experimental techniques do not measure urea–water hydrogen bond interactions directly, but they require an interpretation of the water structure network based on theoretical models with some approximations.

Surfactant formulations are used in a variety of applications, ranging from everyday life consumer products to pharmaceutics [15,16,17]. Addition of electrolytes or polar organic solutes and solvents to aqueous surfactant solutions affect the surfactant self-assembly [18,19,20,21,22,23]. Hence, the effect of additives is important to investigate. A number of studies have been reported regarding the effect of urea and its derivatives on the self-assembly of hydrocarbon-based surfactants or polymers in aqueous solution [24,25,26,27,28,29,30,31]. Two different mechanisms have been proposed to interpret the urea action on aqueous surfactant solutions [24,25]: (i) Indirect mechanism, in which urea acts as a water structure breaker and thus promotes the solvation of hydrophobic solutes, and (ii) direct mechanism, in which urea participates in the solvation of hydrophobic solutes by replacing some water molecules in the solvation layer of the solute. The indirect mechanism of urea action is supported by results from relatively old experimental studies [32,33,34], however, more recent studies [24,25,28] seem to support the direct mechanism. Studies have shown that urea increases the solubility of hydrocarbons (except for methane) in water due to favorable urea–hydrocarbon van der Waals interaction [35,36,37]. Reports on urea effects on sodium dodecyl sulfate (SDS) micellization in aqueous solution have agreed with the direct mechanism of urea action [24,25,28]. Accordingly, the critical micellization concentration (CMC) of SDS in aqueous solution increased upon urea addition [24,25,28], whereas the micelle association number and size decreased upon urea addition. The effect of urea on fluorocarbon solubility in water has, to the best of our knowledge, not been reported.

Fluorinated surfactants have a fluorinated hydrophobic part. The presence of fluorine in the hydrophobic part of surfactants imparts unique properties, such as the ability to repel both oil and water, high chemical and thermal stability, and high wetting ability [38]. Fluorinated surfactants display strong surface activity, greater hydrophobicity, and greater surface tension lowering ability than their corresponding hydrocarbon surfactants due to the weak van der Waals interactions and strong hydrophobicity of fluorocarbon chains compared to hydrocarbon chains [38,39]. Fluorocarbon chains are stiff with larger volume and surface area than hydrogenated ones, thus fluorinated surfactants have a tendency to form assemblies with lower interfacial curvature compared to hydrocarbon surfactants [40]. The unique properties of fluorinated surfactants render them useful in diverse applications like paints, polishes and coatings, stain repellants, lubricants, pesticides, hydraulic fluids, and firefighting foams [38,39,41,42,43,44]. At the same time, the chemical stability of fluorinated surfactants and their incompatibility with hydrocarbon-based materials make them circulate for long times in aqueous media of relevance to the environment and organisms, thus generating great concern [45,46,47,48,49].

The effects of urea on the self-assembly of fluorinated surfactants are little known. To our best knowledge, only one report has been published [50] on urea effects on the fluorinated surfactants lithium perfluorononanoate (LiPFN) and lithium perfluorooctane sulfonate (LiPFOS), and the results suggest differences between hydrocarbon and fluorinated surfactant self-assembly in aqueous urea solutions. An investigation of the differences and similarities of urea effects on fluorinated and hydrogenated surfactant self-assemblies in aqueous solution can offer insights on the mechanism of urea action and its interactions with water and various solutes. An understanding of urea effects on fluorinated surfactant aqueous solution properties is also beneficial from a health and environment perspective. Urea is an essential organic compound synthesized by many organisms, is dissolved into the blood, and is transported and excreted by kidneys through the urine. Urea is also used as a fertilizer in agriculture, and is present in aqueous runoff.

We address here open questions on the effect of urea on the micellization of fluorinated surfactants in aqueous media. We also compare urea effects on fluorocarbon and hydrocarbon surfactants. We focus on ammonium perfluorooctanoate (APFO), which is a widely used perfluorinated surfactant and is highly persistent in environment and biota [45]. The formation and structure of APFO micelles in aqueous urea solutions are assessed on the basis of CMC, degree of counterion dissociation (α), and surface area per headgroup values determined from conductivity and surface tension data. The micellar microenvironment and degree of hydration of APFO in aqueous urea solutions are obtained using pyrene fluorescence and viscosity, respectively.

## 2. Results and Discussion

### 2.1. Micelle Formation and Structure

**Figure 1:**
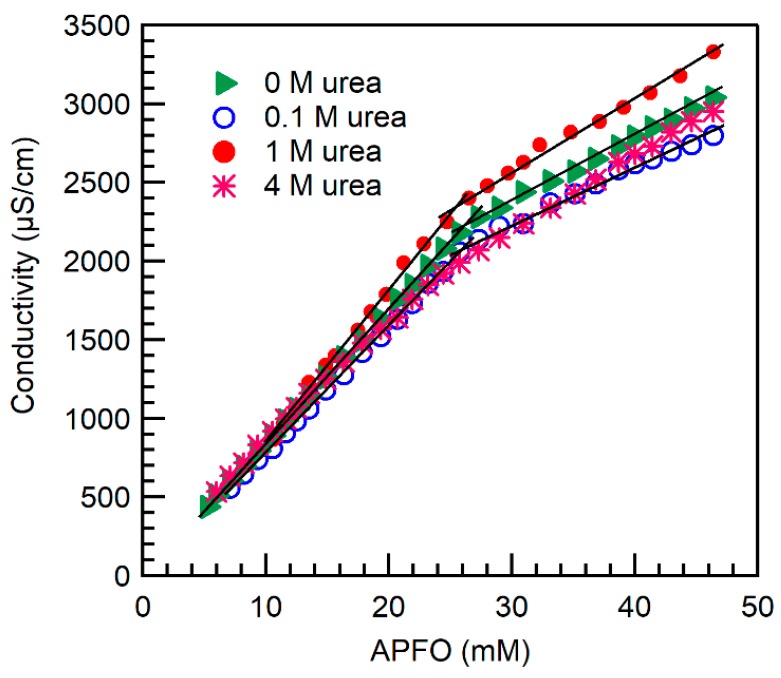
Conductivity of ammonium perfluorooctanoate (APFO) aqueous solutions plotted versus surfactant concentration in the absence and in the presence of added urea at various concentrations (shown inside the graph) (24 °C). Linear fits to the data points below and above the break point have been applied in order to determine the critical micelle concentration (CMC).

The conductivity of aqueous APFO solutions as a function of surfactant concentration in the absence and in the presence of urea is shown in Figure 1. The change in the slope at the CMC becomes more gradual with an increase in urea concentration. The CMC and the degree of counterion dissociation (α) (refer to Section 3.2) of APFO in aqueous solution in the absence and in the presence of added urea are reported in Table 1. Upon addition of urea, the CMC of APFO in aqueous solution decreased and the degree of APFO counterion dissociation (α) increased. The variation of CMC and α of APFO in aqueous solution in the presence of urea has not been previously reported in the literature.

**Figure 2:**
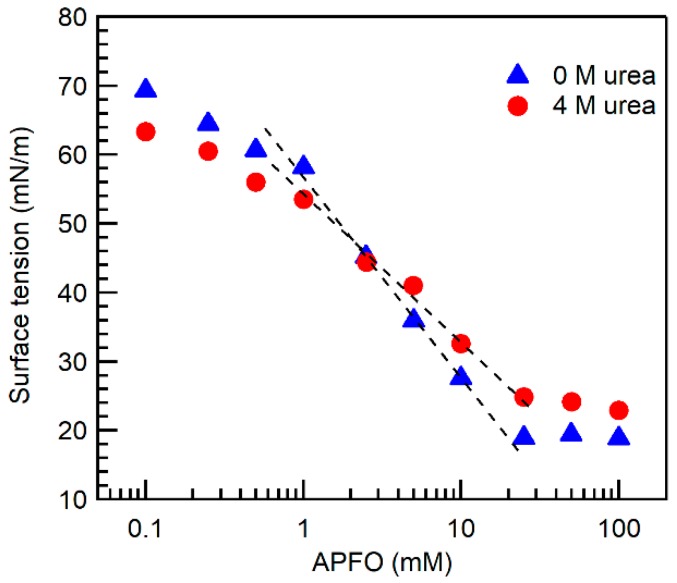
Surface tension of APFO aqueous solutions in the absence and in the presence of 4 M urea (24 °C). Linear fits have been applied to the data points where the surface tension decreases prior to reaching the CMC.

The surface tension of APFO aqueous solution in the absence and in the presence of urea is plotted in Figure 2 as a function of surfactant concentration. The surface tension of APFO solutions above the CMC reached a constant value of ~18 mN/m in the absence of urea, and ~23 mN/m in the presence of 4 M urea. No surface tension data of APFO aqueous solutions in the presence of urea are available in the literature. From the surface tension plots we determined the slope dγ/dlogC at the CMC, which can be used to estimate the maximum surface excess concentration, $\Gamma_{\max}$ [52,53,54]:

(1) $$\Gamma_{max}=-\frac{1}{2.303nRT}{\left(\frac{d\gamma }{dlogC}\right)}_{T,P}$$

where γ is the surface tension, R the universal gas constant (8.314 J mol^−1^ K^−1^), T the absolute temperature, and C the surfactant concentration in mM [52]. The constant *n* is taken as 2 for ionic surfactants in which the surfactant ion and counterion are monovalent [52].

For APFO aqueous solution in the absence of urea, the slope (dγ/dlogC) determined at the CMC is −29.6 mN/m, and $\Gamma_{\max}$ calculated from Equation (1) is 2.6 × 10^−10^ mol cm^−2^. In the presence of 4 M urea, dγ/dlogC = −21.5 mN/m and $\Gamma_{\max}$ = 1.9 × 10^−10^ mol cm^−2^.

The minimum area occupied by a surfactant molecule (A_min_) at the air/liquid interface can be calculated from the maximum surface excess concentration $\Gamma_{\max}$ [52,53,54]:

(2) $$A_{min}=\frac{1}{N\Gamma_{max}}$$

where N = 6.022 × 10^23^ is the Avogadro number.

The A_min_ values thus obtained for APFO in aqueous solution in the absence and in the presence of 4 M urea are 64 ± 2 and 88 ± 5 Å^2^, respectively. This 40% increase upon addition of 4 M urea indicates that APFO molecules pack relatively loosely in the presence of urea.

The tendency of amphiphiles to assemble into specific structures can be informed by the critical packing parameter (CPP) which is defined as:

(3) $$CPP=\frac{V_{o}}{A_{min}l_{c}}$$

where V_0_ is the volume of the surfactant hydrophobic chain (tail volume) and $l_{c}$ is the extended length of the surfactant hydrophobic chain (tail length) [15,55].

The volume (V_o_) and length ($l_{c}$) of the APFO fluorocarbon tail were taken from Blanckschtein’s molecular model, developed to evaluate the free energy of micellization of fluorocarbon surfactants [56]. The reported volumes of the CF_2_ and CF_3_ groups are 41.6 and 84.0 Å^3^, respectively [56]. A linear-chain perfluorinated surfactant with n_c_ carbon atoms in the tail will have (n_c_ − 1) CF_2_ groups and one CF_3_ group. Therefore, the volume (in Å^3^) of a fluorinated surfactant tail with n_c_ carbon atoms is $V_{f,c}=\left[41.6\;\left(n_{c}-1\right)+84\right]$ which is equal to:

(4) $$V_{f,c}=\left[41.6n_{c}+42.4\right]$$

The extended length (in Å) of a fluorocarbon chain with n_c_ carbon atoms can be given as [56]:

(5) $$l_{f,c}=\left[1.3n_{c}+2.04\right]$$

The volume and extended length of an APFO fluorocarbon chain calculated from the above equations are 333.6 and 11.14 Å, respectively. The calculated CPP values (equation 3) of APFO in aqueous solution in the absence and in the presence of 4 M urea are 0.47 and 0.34, respectively. The CPP value of 0.47 falls in between 0.33 and 0.5 (0.33 < CPP < 0.5) which suggests that APFO micelles formed in aqueous solution have a tendency to be cylindrical in shape [15], consistent with small-angle neutron scattering (SANS) on 120 mM APFO in D_2_O [57]. With the addition of 4 M urea, the CPP of APFO in aqueous solution decreased by about 30% to a value (0.34) that is close to the CPP range of spherical micelles (≤0.33), which suggests that APFO micelles would tend to become spherical in the presence of urea. The increase in the surface area per APFO headgroup and the decrease in the packing density at the micelle surface upon urea addition suggest the localization of urea in APFO micelles. If there were localization of urea in the interior of the APFO micelles, then there should be a change in the micelle microenvironment. The micellar microenvironment is probed here with pyrene fluorescence.

### 2.2. Micellar Microenvironment

**Figure 3:**
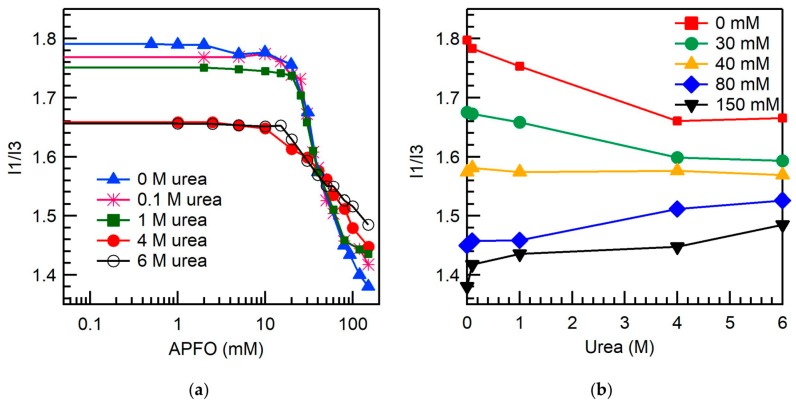
(**a**) Pyrene fluorescence intensity I1/I3 ratio of APFO aqueous solutions plotted versus surfactant concentration in the absence and in the presence of urea (at various concentrations shown inside the graph) (22 °C); (**b**) variation with urea concentration of the pyrene fluorescence intensity I1/I3 ratio of APFO at various concentrations (shown inside the graph) in aqueous solution. The lines connecting the data points are meant as guides to the eye.

Figure 3a shows the pyrene fluorescence intensity I1/I3 ratio (refer to Section 3.4) of APFO aqueous solutions in the absence and in the presence of added urea, plotted as a function of surfactant concentration. Above the CMC, the I1/I3 ratio decreased due to the partition of pyrene from the polar aqueous solution to the hydrophobic APFO micellar environment. Figure 3b shows the variation of pyrene fluorescence intensity I1/I3 ratio of APFO aqueous solutions with urea concentration. Below the CMC, where pyrene molecules are located in an aqueous environment, the I1/I3 ratio of APFO aqueous solutions decreases with urea concentration, reflecting the greater hydrophobicity of urea-water solutions (see I1/I3 ratio for 0 mM APFO) compared to plain water. In micellar aqueous APFO solutions, pyrene molecules are expected to locate at the outer palisade layer of the APFO micelles [58]. At the CMC and slightly above the CMC of APFO, the I1/I3 ratio decreased with urea concentration. This is because the APFO micelles formed in the vicinity of CMC may incorporate appreciable water content and, upon urea addition, urea molecules may localize at the palisade layer of APFO micelles where pyrene molecules are also present, replacing some water molecules. This decreases the polarity of pyrene’s microenvironment in APFO micelles. At high APFO concentrations, the I1/I3 ratio is increasing with urea concentration. This is because APFO micelles have lower water content (decrease in hydration) at higher surfactant concentrations compared to micelles formed near the CMC [59]. Urea partitioning into APFO micelles increases the surface area per headgroup of the APFO micelles, and pyrene that was localized in the palisade layer of APFO micelles moves into a more aqueous environment, increasing the I1/I3 ratio. The I1/I3 ratio at 80 mM and 150 mM APFO increased by 5% and 8%, respectively, with the addition of 6 M urea. At intermediate APFO concentration (40 mM), i.e., nearly twice that of CMC, the I1/I3 ratio remained invariant with urea concentration. This may be the result of two opposing factors: Urea localization in APFO micelles, where pyrene is also located by replacing some water molecules, decreases the I1/I3 ratio; urea localization in APFO micelles causes pyrene to move outwards into a more aqueous environment, thus increasing the I1/I3 ratio.

The variation of the I1/I3 ratio of APFO aqueous solutions with urea concentration suggests that the apparent hydrophobicity of aqueous solution may increase with urea addition; the increase or decrease in the micropolarities of APFO micelles with urea addition will depend on the added urea concentration and APFO concentration. The change in micropolarities and the increase in surface area per headgroups of APFO micelles with urea addition suggest urea localization in APFO micelles replacing some water molecules, and this decreases the surfactant packing density at the micelle surface affecting the micelle structure. 

**Figure 4:**
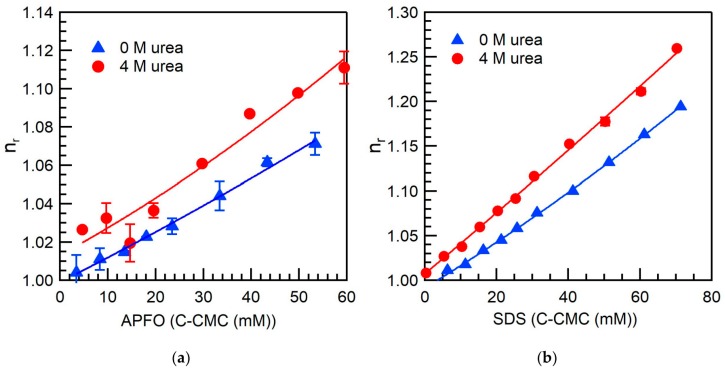
Relative viscosity, *η_r_*, of APFO (**a**) and SDS (**b**) aqueous solutions in the absence and in the presence of 4 M urea, plotted as a function of micellized surfactant concentration. The lines through the viscosity data points are fits to Equation (6).

While direct information on the variation of the APFO micelle size with urea addition is not available in the literature, indirect evidence on the micelle size/shape is obtained here through analysis of viscosity data. Figure 4 shows the relative viscosity (refer to Section 3.5) of APFO aqueous solutions plotted as a function of micellized surfactant concentration, in the absence and in the presence of urea. Such viscosity data have not been previously reported in the literature. The relative viscosity can be expressed in terms of the volume fraction occupied by micelles (φ) [60,61]:

(6) $$\eta_{r}=\frac{\eta }{\eta_{o}}=1+\nu \phi +k_{1}{\left(\nu \phi \right)}^{2}+O{\left(\phi \right)}^{3}$$

where ν is a shape factor reflecting the micelle shape, and $k_{1}$ is a coefficient corresponding to pair-wise hydrodynamic interactions between micelles [61]. For polydisperse micellar solutions, ν represents the shape of the average micelle [61]. The term $k_{1}{\left(\nu \phi \right)}^{2}$ accounts for hydrodynamic interactions, and the term $O{\left(\phi \right)}^{3}$ arises due to direct micelle-to-micelle interactions and can be neglected for dilute surfactant solutions [60,61]. The volume fraction of micelles (ϕ) includes the water of hydration associated with micelles, and is given by $\phi =V_{s}^{hyd}\left(c_{s}-c_{1}\right)$, where $V_{s}^{hyd}$ is the hydrated volume of a surfactant molecule, $c_{s}$ is the total surfactant concentration, and $c_{1}$ is the concentration of unassociated surfactant (taken to be the CMC) [61]. $V_{s}^{hyd}$ is calculated from the x-coefficient value obtained by fitting a second degree polynomial equation (Equation (6) neglecting the term $O{\left(\phi \right)}^{3}$) to the experimental viscosity data set given in Figure 4. For spherical micelles, ν = 2.5 [60,61]. For cylindrical micelles, the shape factor ν is given by [60]:

(7) $$\nu =2.5+0.407{\left(J-1\right)}^{1.508},\;\mathrm{for}\;1\;<\;J\;<\;15$$

(8) $$\nu =1.6+\frac{J^{2}}{15}\left[\frac{1}{\left(\ln\left(2J\right)-1.5\right)}+\frac{3}{\left(\ln\left(2J\right)-0.5\right)}\right],\mathrm{for}\;J\;>\;15$$

(9) $$J=\left(\frac{L}{d}\right){\left[\frac{2}{3-\left(d/L\right)}\right]}^{1/2}$$

where (L/d) is the axial ratio of a cylindrical micelle, L is the length of a cylindrical micelle including contributions from the two hemispherical ends, and d is the micelle diameter [60]. For the purpose of hydrodynamic calculations, a cylindrical rod of axial ratio (L/d) can be equated to a prolate ellipsoid of equal length and volume [60]. The axial ratio (J) of such an equivalent prolate ellipsoid is given in Equation (9).

For APFO in water (no urea), we estimated the shape factor ν from Equation (7) to be 2.97, using the axial ratio (L/d) value obtained from a SANS study of 120 mM APFO in aqueous NH_4_Cl-NH_4_OH buffer, pH 8.8, ionic strength = 0.1 [57]. The hydrated volume of an APFO molecule ($V_{s}^{hyd}$) estimated from the relative viscosity Equation (6) is 0.710 nm^3^. The total volume of water that is hydrating an APFO molecule = 0.334 nm^3^ is obtained by subtracting the molecular volume of APFO (0.376 nm^3^) [62] from $V_{s}^{hyd}$. From the volume of a single water molecule (0.030 nm^3^), we estimated the number of water molecules hydrating an APFO molecule to be 11. Since the size and shape of APFO micelles in aqueous urea solutions are not available in the literature, we considered two extreme cases to estimate $V_{s}^{hyd}$ for APFO in aqueous urea solutions: (I) Axial ratio and shape factor ν of APFO micelles the same as those in plain water, i.e., APFO micelles are cylindrical, and (II) APFO micelles are spherical (ν = 2.5) (consistent with CPP = 0.34 estimated from our surface tension data). In case (I), the hydrated volume of an APFO molecule ($V_{s}^{hyd}$) in 4 M urea solution is estimated to 0.770 nm^3^, a ~10% increase compared to APFO in plain water. In case (II), $V_{s}^{hyd}$ in 4 M urea solution is estimated to 0.910 nm^3^ (~30% increase). The hydrated volume per APFO molecule increases significantly in both cases considered. The hydrated volume of surfactant molecules thus calculated for aqueous urea solution would also include any urea molecules strongly solvating the surfactant molecules. Assuming the number of water molecules hydrating an APFO molecule in the case of 4 M urea to be the same as in plain water, the observed increase in $V_{s}^{hyd}$ corresponds to the urea molecules solvating each surfactant molecule. Subtracting the molecular volume of an APFO molecule (0.376 nm^3^) [62] and the volume of water molecules hydrating an APFO molecule (0.334 nm^3^) from $V_{s}^{hyd}$ gives the total volume of urea solvating an APFO molecule to be equal to 0.064 nm^3^ for case (I), and to 0.204 nm^3^ for case (II). From the volume of a single urea molecule (0.075 nm^3^) [63], we can estimate the average number of urea molecules solvating an APFO molecule to be 0.9 in case (I) and 2.7 in case (II).

### 2.3. Comparison of Fluorocarbon and Hydrocarbon Surfactants

The CMC of APFO in aqueous solution decreased by 23% and the degree of counterion dissociation (α) increased by 19% with the addition of 4 M urea, but no considerable change in the CMC and degree of counterion dissociation (α) was observed at low (0.1 M) urea concentrations. Urea can affect surfactant micellization through water-structure modification, changes in electrostatic charges and hydrophobic interactions. As discussed in the Introduction, recent molecular simulation studies have shown that urea has a negligible effect on the water structure network. Therefore, we believe that the modulation in the surfactant CMC with urea addition is mainly due to changes in electrostatic and hydrophobic interactions. Addition of 6 M urea increases the dielectric constant of water from 78 to 92 [31]. According to Coulomb’s law, the increase in the medium dielectric constant should decrease the force between charges. Consequently, addition of urea to aqueous APFO solution should (i) decrease the attraction between headgroup COO⁻ and counterion NH_4_⁺ due to which counterion dissociation increases, and (ii) decrease the repulsion between COO⁻ headgroups; both (i) and (ii) favor micellization. The decrease in headgroup–headgroup repulsion should decrease the surface area per headgroup but, on the contrary, the surface area of APFO is found to increase with urea addition due to urea localization. Studies have shown that urea can decrease the hydrophobic effect due to which the aqueous solubility of hydrocarbons increases [35,36,37,64]. In urea–water solution, the enthalpy of cavity formation due to hydrocarbons decreases due to the energy regained from interactions (van der Waals, dipole-dipole, and hydrogen bond) between urea and hydrocarbons, and this increases the hydrocarbon solubility [35,36,37,64]. The effect of urea on fluorocarbon solubility in aqueous solutions is not reported in the literature. Fluorocarbons are weakly susceptible to fleeting dipoles and have low intermolecular attractive forces and weak van der Waals interactions due to fluorine atom low polarizability [38]. Because of this, we believe that the decrease in the hydrophobic effect due to urea–fluorocarbon interactions should be lower compared to hydrocarbon chains, and the aqueous solubility of fluorocarbon chains should not increase much with urea addition. The decrease in the hydrophobic effect should oppose micellization. The decrease in the CMC of APFO with urea addition may be due to the combined effect of urea on electrostatic and hydrophobic interactions.

For better understanding of urea effects on APFO micellization in aqueous solution, we compare our results on APFO with results on other surfactants. Table 1 summarizes the CMC and the degree of counterion dissociation (α) of APFO (our results), lithium perfluorononanoate (LiPFN), lithium perfluorooctane sulfonate (LiPFOS) [50], and SDS (our results and literature [24,27,28]). Similar to APFO, the CMC decreased and the degree of counterion dissociation (α) increased for the fluorinated surfactants LiPFN and LiPFOS with urea addition, on the basis of conductivity and fluorescence probe (1-anilinonaphthalene-8-sulfonate, auramine, and pyrene) data [50]. The CMC decrease upon urea addition has been ascribed to the replacement of some water molecules by urea in fluorinated surfactant micelles which favors the formation of micelles [50]. This urea effect on the self-assembly of fluorinated surfactants is consistent with the direct mechanism of urea action [50].

Contrary to what is observed in fluorinated surfactants, the CMC of hydrocarbon surfactants increases with urea addition to water [24,28]. Our conductivity results (Figure S1) show the CMC of SDS in aqueous solution did not change much at low urea concentrations, but increased by 11% in the presence of 4 M urea. This increase is in agreement with literature and is ascribed to an increase of the aqueous solubility of the surfactant hydrocarbon chains [24,28]. We believe that in the case of SDS, the urea effect on hydrophobic interactions opposing micellization is dominating over the urea effect on electrostatic interactions favoring micellization; hence the CMC of SDS is increasing with urea addition. The CMC trends for fluorinated surfactants and SDS in aqueous solution with urea addition are opposite, whereas the degree of counterion dissociation (α) increased with urea addition for both fluorinated surfactants and SDS. The difference in the CMC trends between fluorocarbon surfactants and hydrocarbon surfactants with urea concentration is not emanating from the interaction of urea with the polar headgroup of the surfactant; this can be ascertained from the CMC trends of lithium 1H,1H,2H,2H–perfluorodecyl sulfate (LiHFDeS) and lithium dodecyl sulfate (LiDS), where the CMC of LiHFDeS remained almost constant, but the CMC of LiDS increased with urea concentration [50]. To further probe the differences in urea effects between fluorocarbon surfactant and hydrocarbon surfactant self-assembly, we consider next the micelle structure and microenvironment.

The effect of urea on the APFO micelle structure can be inferred from our surface tension results, which show the surface area per APFO molecule to increase by 40% with 4 M urea addition. This increase suggests localization of urea at the APFO micelle surface, and this is supported by our pyrene fluorescence results. The increase in the surface area per APFO headgroup also reduces the headgroup–headgroup repulsions and surface charge density. This may also be a reason for the observed increase in the degree of counterion dissociation of APFO with urea addition. The decrease in the CPP of APFO from 0.47 to 0.34 with 4 M urea addition suggests a change of micelle shape from cylindrical to spherical.

The effects of urea addition on fluorinated surfactant micelle structure and association number have not been previously reported, however, information on effects of urea on the SDS micelle structure is available [24,28,29]. Addition of 6 M urea resulted in a 30% decrease in the SDS micelle association number, 11% decrease in the micelle radius, and 13% increase in the surface area per headgroup [24]. However, the increase in surface area per SDS headgroup is much lower [24,29] (8%) compared to that of APFO (40%) for the same urea concentration (4 M). The decrease in the packing density with urea addition is also lower in the case of SDS when compared to APFO. The CPP of SDS decreased from 0.34 to 0.31 (9%) with 6 M urea addition [24], whereas, in the case of APFO reported here, the CPP decreased from 0.47 to 0.34 (28%) with 4 M urea addition.

**Figure 5:**
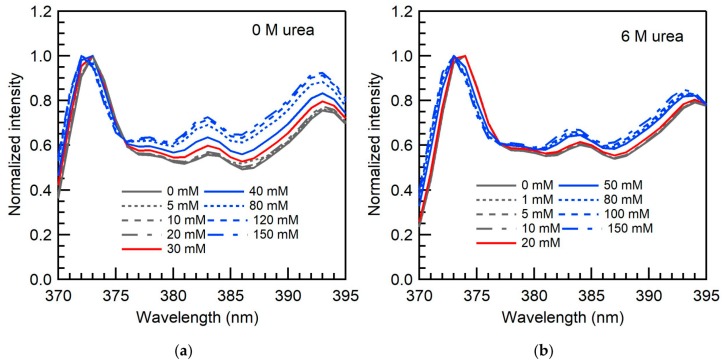
Pyrene monomer emission spectra of APFO aqueous solutions in the absence (**a**) and in the presence of urea (**b**). The spectra have been normalized by dividing the intensity at each wavelength with the intensity of the first peak (I1). The grey lines (——, ·········, - - - - -, — - — - ) indicate the spectra of pyrene for APFO concentrations below CMC, the red line (——) is for APFO concentration near CMC and the blue lines (——, ·········, - - - - -, — - — - ) are for APFO concentrations above CMC.

The micropolarities sensed by pyrene in APFO micelles at APFO concentrations near the CMC decrease with the addition of urea due to the localization of urea molecules in APFO micelles where pyrene is also located. At high APFO concentrations, the micropolarity sensed by pyrene increases with urea concentration, and this may be because of pyrene moving outwards into a more aqueous environment with the localization of urea at APFO micelles. At APFO concentration nearly twice that of the CMC, the micropolarity in the presence of urea appears the same as in plain water, as a result of possibly the two opposing effects discussed previously: (i) Urea localizes in APFO micelles where most pyrene is located and decreases micropolarity, and (ii) urea localization in APFO micelles causes some pyrene to move outwards into more aqueous environment, which increases the micropolarity sensed by this pyrene. The pyrene monomer emission in APFO aqueous solutions shows a spectral shift and a change in the shape of normalized spectra around the CMC (Figure 5). Below CMC, the spectra do not change shape with APFO concentration because the fluorescence intensity emanates from the contribution of only free pyrene. However, above the CMC, a change in the shape of spectra is observed because the fluorescence intensity includes contributions from both free and micelle-bound pyrene [65]. As the urea concentration increases, the shape of normalized spectra above and below the CMC do not change much, because the intensity contribution from micelle-bound pyrene decreases as some pyrene moves outwards into a more aqueous environment (the intensity of the peak at 383 nm (I3) decreases).

For LiPFN at 20 mM (almost double its CMC (11.3 mM)), the pyrene I1/I3 values in the presence of urea were almost identical with those in plain water [50], similarly to our fluorescence results for APFO concentration (40 mM) nearly twice its CMC. However, our results demonstrate that at lower and at higher APFO concentrations the micropolarities sensed by pyrene localized in APFO micelles changed with urea concentration.

For SDS aqueous solutions, the I1/I3 ratio decreased below CMC and increased above CMC with urea concentration (Figure S2). The concentration of pyrene (<0.001 mM) added to the surfactant solution is very low, and we expect a maximum one pyrene molecule per SDS micelle [66]. With the addition of urea, the urea molecules localize in SDS micelles, increasing the surface area per headgroup and causing the pyrene molecules to move outwards into a more aqueous environment. A decrease in the I1/I3 ratio with urea concentration in the vicinity of CMC is not observed in the case of SDS, unlike APFO, because for SDS the CMC is increasing with urea concentration and pyrene is located in the hydrophobic interior of SDS micelles and not at the palisade layer. Normalized monomer emission spectra of pyrene in SDS aqueous solutions (Figure S3) show a change in the shape of spectra above CMC due to the contribution of fluorescence intensity from both free and micelle-bound pyrene. The difference in normalized intensities of pyrene spectra below and above the CMC of SDS decreases slightly with urea concentration due to the displacement of bound pyrene from the micelle core towards the outer region of the micelle. Similar to our results, an increase in the pyrene I1/I3 ratio of SDS aqueous solutions above CMC (at 20 mM) with added urea has been previously reported [24]. Electron spin resonance and fluorescence using 8-anilino-1-naphthalensulfonic acid (ANS), rhodamine B (RB), or auramine O (AuO) have shown the micropolarity to decrease and the microviscosity to increase with urea addition for SDS micelles in aqueous solution [24,25].

The viscosity results reported here show that the hydrated volume per APFO molecule increased by ~10–30% with 4 M urea addition due to the localization of urea molecules in APFO micelles. The hydrated volume of a SDS molecule ($V_{s}^{hyd}$) in water and in 4 M urea solution estimated from fitting Equation (6) to the data of Figure 4 (bottom) are 1.630 and 2.240 nm^3^, a 37% increase (the SDS micelles formed in aqueous urea solutions are spherical [24] hence we considered a shape factor of 2.5). From the volume of a SDS molecule (0.410 nm^3^) [67], the hydrated volume of a SDS molecule ($V_{s}^{hyd}$) in water (1.630 nm^3^), and the volume of a single water molecule (0.030 nm^3^), we estimated the number of water molecules hydrating a SDS molecule to be 40, which is 10 times that of APFO. This big difference in hydration between hydrocarbon and fluorocarbon surfactant merits further investigation. Assuming the number of water molecules hydrating an SDS molecule in the case of 4 M urea to be the same as in plain water, the increase in $V_{s}^{hyd}$ corresponds to urea molecules solvating each surfactant molecule. Subtracting the volume of one SDS molecule (0.410 nm^3^) [67] and the volume of the water molecules hydrating one SDS molecule (1.200 nm^3^) from $V_{s}^{hyd}$, gives the total volume of urea solvating one SDS molecule = 0.630 nm^3^. From the volume of a single urea molecule (0.075 nm^3^) [63], we estimated the average number of urea molecules solvating a SDS molecule to be 8.4. The number of urea molecules solvating each SDS molecule is much higher than that for APFO (0.9–2.7).

## 3. Materials and Methods

### 3.1. Materials

Ammonium perfluorooctanoate (C_7_F_15_COONH_4_, CAS number: 3825-26-1, MW = 431.1 g/mol), also known as pentadecafluorooctanoic acid ammonium salt or perfluoro-n-octanoic acid ammonium salt, was obtained from SynQuest Laboratories (98% purity, Alachua, FL, USA) and used as received. Sodium dodecyl sulfate (C_12_H_25_SO_4_Na, MW = 288.4 g/mol), also known as sodium lauryl sulfate, was obtained from Sigma Life Science (≥98.5% purity, St. Louis, MO, USA). Urea (NH_2_CONH_2_), 99% pure, was obtained from Alfa Aesar (Haverhill, MA, USA). All samples were prepared using milli-Q purified water. The samples were allowed sufficient time to equilibrate following the mixing of ingredients.

We selected in this study ammonium perfluorooctanoate since it is a fluorocarbon surfactant that has been widely used and is highly persistent in the environment and biota [68]. We selected the hydrocarbon surfactant SDS to compare to APFO since (i) both are anionic surfactants, (ii) both have linear alkyl chains, (iii) both surfactants fully dissociate below the CMC [69,70], (iv) SDS is very well studied, and (v) some solution properties of fluorinated surfactants are found to be comparable to properties of hydrocarbon surfactants having alkyl chain length 1.5 times that of fluorinated surfactants, e.g., the 8 CF_2_ groups of APFO are equivalent to the 12 CH_2_ groups of SDS [42,71].

### 3.2. Conductivity

Addition of ionic surfactant to an aqueous solution causes the number of ions to increase, and results in an increase in the conductivity of the solution. Below the CMC, the surfactants dissociate into ions and exist as un-associated molecules in aqueous solution. Above CMC, the surfactants form micelles in solution (with counterions binding onto the micellar surface forming electrical double layer), which are in equilibrium with unassociated surfactant of concentration approximately equal to the CMC. Above the CMC, the relative increase in the solution conductivity with surfactant concentration is lower compared to that below the CMC. This is because a fraction of counterions is attracted to the micelles forming electrical double layer and, hence, the total number of charge carriers is lower than the apparent surfactant concentration, and the conductivity vs. surfactant concentration curve shows a break point at the CMC. This break point can be used to determine the CMC [72]. The ratio of the conductivity vs. surfactant concentration slopes above (S_2_) and below (S_1_) the CMC gives the degree of counterion dissociation (α = S_2_/S_1_) [51].

Accumet XL 50 and Model 20 conductivity meters (Fisher Scientific, Hampton, NH, USA) with potassium chloride electrodes were used to measure the conductivity of aqueous surfactant solutions. Temperature corrections were taken into account while standardizing the conductivity meter for accurate readings. The conductivity of aqueous APFO solutions in the absence and in the presence of urea was measured at 24 (±2 °C) in the APFO concentration range 0–50 mM.

### 3.3. Surface Tension

When surfactant is added to an aqueous solution, the surfactant molecules accumulate at the air-water interface and decrease the surface tension. The surface tension decreases and reaches an almost constant value or changes with a much lower slope (plateau-like region) above the CMC. This is because, following the formation of micelles at the CMC, the chemical potential of the surfactant hardly changes and, consequently, conditions do not change at the air–water interface. The slope of the surface tension vs. logarithm of surfactant concentration plot (dγ/dlogC) determined at the CMC can be used to estimate surface properties such as the maximum surface excess concentration $\Gamma_{\max}$ and the minimum area occupied by a surfactant molecule (A_min_) at the air/liquid interface [52,53,54].

The surface tension of aqueous APFO solutions in the absence and in the presence of urea was measured by a Wilhelmy plate using a Krüss (Hamburg, Germany) model K100 tensiometer. The surface tension of 4 M urea aqueous solution in the absence of surfactant is found 68 mN/m.

### 3.4. Micropolarity

Pyrene fluorescence spectroscopy is used here to probe the micropolarity of aqueous surfactant solutions. Pyrene is hydrophobic, and it tends to partition from the aqueous phase to a hydrophobic environment. The emission spectrum of pyrene monomer exhibits a vibronic fine structure and the ratio of the intensities of first and third vibronic peaks (I1/I3) strongly depends on polarity of its microenvironment [18,73]. The changes in the microenvironment characteristics of APFO micelles with urea addition can be assessed by observing the I1/I3 ratio as a function of urea concentration. There are no uniform criteria for obtaining CMC values from I1/I3 vs. surfactant concentration plots, and different authors follow different methods: The first turning point in the I1/I3 vs. surfactant concentration curve [58]; the inflection point in the ratio of peak intensities of fine structure (I1/I3, I3/I1, or I5/I1) vs. surfactant concentration curve [74,75]; the intersection of straight lines fitted to the first plateau region below CMC and the linear part of the I1/I3 decrease region (near the inflection point) or the linear part of I1/I3 decrease region (near the inflection point) and second linear plateau region above CMC (not observed in the case of our APFO system) in I1/I3 vs. surfactant concentration curve [75]. For the fluorinated surfactants lithium perfluorononanoate (LiPFN) and sodium perfluorononanoate (NaPFN), the CMC has been determined from the concentration corresponding to maximal changes that is obtained from the derivative of d(I1/I3)/d(surfactant concentration) plots [76]. For the APFO systems in this study, the concentration value obtained from the intersection of straight lines fitted to the first plateau region of the I1/I3 vs. surfactant concentration curve and to the linear part of I1/I3 decrease region (near the inflection point) provided CMC values that are consistent with those obtained from conductivity.

Pyrene purchased from Fluka (Buchs, Switzerland) was used as a probe of micropolarity. Two μL of 1 mM pyrene in ethanol (ACS/USP grade) was added to 3 g sample solutions for fluorescence spectroscopy. The resulting overall pyrene and ethanol concentrations were about 0.7 μM and 6.7 × 10^−4^ vol %, respectively. Pyrene fluorescence emission spectra of aqueous APFO solutions in the range 1–200 mM in the absence and in the presence of urea were recorded at 22 °C using a Hitachi model F-2500 fluorescence spectrophotometer (Stoughton, MA, USA). The vibrational fine structure of pyrene did not change in the presence of urea, and only changes in the intensity of vibronic bands were observed. This suggests that no specific interaction between the fluorescence probe and urea takes place [24].

### 3.5. Viscosity

The viscosity of surfactant solutions is related to the micelle size and shape, volume fraction, and hydration [59,77]. We measured the viscosity of aqueous surfactant solutions using Cannon-Fenske capillary viscometers of sizes 50 and 100 (depending on the viscosity range of the sample solutions). The efflux time for each sample concentration was recorded, and two consecutive readings with the same sample concentration inside with time difference less than 0.50 s were considered. The temperature of solutions during viscosity measurements was 20 °C controlled to within ±0.5 °C by placing the viscometer inside a thermostated water bath. The efflux times were reproducible to ± 0.2 % (each measurement was repeated 3 times) and were measured with an accuracy of ±0.5 sec. The kinematic viscosity (*η*) is calculated by multiplying the efflux time with the viscometer calibration constant (provided by the manufacturer, Cannon Instrument Co, State College, PA, USA). The ratio of the kinematic viscosity of the solution (*η*) and the kinematic viscosity of pure solvent ($\eta_{o}$) gives the relative viscosity ($\eta_{r}=\eta /\eta_{o}$) of the solution.

## 4. Conclusions

The origins of urea effects on aqueous hydrocarbon surfactant micellization are still not resolved, whereas for fluorocarbon surfactants they are largely unknown. We investigated the effect of urea on the micellization of ammonium perfluorooctanoate (APFO) in aqueous solution. The results in this study are consistent with the direct mechanism of urea action on APFO micellization. The combined effect of urea on electrostatic and hydrophobic interactions influences the self-assembly, as attested by the change in CMC upon urea addition: the CMC of APFO decreased with urea addition. Urea also affects the APFO micelle structure, as it localizes in micelles and tends to change the micelle shape. Urea localization in APFO micelles is supported by the observed changes in surface area per APFO headgroup, hydrated volume of surfactant molecule, packing density at micelle surface, and micropolarity of APFO micelles. Upon 4 M urea addition, the surface area per APFO headgroup increased by about 40%, the hydrated volume of a surfactant molecule $V_{s}^{hyd}$ increased by ~10–30%, and the CPP decreased from 0.47 to 0.34.

This is the first study to report on urea effects on surface area per headgroup, packing density, possible shape changes, and hydration of fluorinated surfactant micelles. There is only one other report [50] on urea effects on the CMC and counterion binding in fluorinated surfactants. The results presented here show that urea effects on fluorinated and hydrocarbon surfactant micellization have significant differences, such as modulation in the CMC and micelle structure. The CMC of the fluorocarbon surfactant APFO in aqueous solution decreased with increasing urea concentration, whereas, in the case of the hydrocarbon surfactant SDS, the CMC increased [24,25,28]. Urea affected the SDS micelle structure by increasing the surface area per headgroups and decreasing the micelle association number, however, the increase in surface area of SDS (8% for 4 M urea) [24] is much lower compared to that of APFO (40% for 4 M urea). Unlike the case of APFO, urea addition to aqueous SDS solution did not induce any micelle shape changes [24]. The differences in the effect of urea on APFO and SDS may be due to the differences in urea–fluorocarbon and urea–hydrocarbon interactions in water. An investigation of molecular interactions between urea and fluorocarbon or hydrocarbon chains in aqueous solution and their effect on thermodynamic parameters (enthalpy, entropy) can be informative to this end; this is beyond the scope of the present work.

Beyond the physicochemical fundamentals of interest to this study, the association or partitioning of fluorinated surfactants in the presence of urea has a broader impact, since fluorinated surfactants are highly persistent in the aqueous environment and in various organisms where urea or related compounds may also be present. Further, fluorinated surfactants can be used in applications such as solubilization and purification of membrane proteins in which urea is used as a chaotropic agent [78].

## Figures and Tables

**Table 1:** CMC and degree of counterion dissociation (α) of APFO, lithium perfluorononanoate (LiPFN), lithium perfluorooctane sulfonate (LiPFOS), and sodium dodecyl sulfate (SDS) in aqueous solution, in the absence and in the presence of added urea. The data for APFO and SDS were determined in the present study, and compared with LiPFN, LiPFOS, and SDS data from the literature [24,27,28,50,51].

Surfactant	Urea Concentration (M)	Temperature (°C)	CMC (mM)	α
APFO	0	24	26.5 (±0.1) ^a^,24.5 (±0.7) ^b^, 25 [51]	0.47, 0.40 [51]
APFO	0.1	22	27.4 (±0.3) ^a^, 22.5 (±2) ^b^	0.45
APFO	1	22	25.5 (±0.5) ^a^, 22.5 (±0.2) ^b^	0.45
APFO	4	24	20.6 (±0.3) ^a^, 20 (±0.2) ^b^	0.56
APFO	6	22	15.5 (±0.5)^b^	-
LiPFN	0	25	11.3 [50]	0.57 [50]
LiPFN	3	25	9.5 [50]	0.70 [50]
LiPFN	6	25	9.3 [50]	0.71 [50]
LiPFN	8	25	10.0 [50]	0.81 [50]
LiPFOS	0	25	7.1 [50]	0.63 [50]
LiPFOS	3	25	5.8 [50]	0.77 [50]
LiPFOS	6	25	5.2 [50]	0.90 [50]
SDS	0	24	8.7 (±0.2) ^a^, 9(±1) ^b^, 8.2 [24]	0.41, 0.30 [28]
SDS	0.1	22	7.5 ^c^ [27]	0.48 ^c^ [27]
SDS	1	24	8.6 (±0.1) ^a^, 8.5 (±1) ^b^, 8.75 ^c^ [27]	0.45, 0.43 ^c^ [27]
SDS	4	24	10 (±0.3) ^a^, 10.5 (±1) ^b^, 9.6 [28], 10.5 [24]	0.64, 0.40 [28]

^a^ CMC determined from conductivity, ^b^ CMC determined from pyrene fluorescence, ^c^ CMC and α determined at 30 °C.

## Supplementary Materials

The following are available online at https://www.mdpi.com/1422-0067/20/22/5761/s1.
