# Supplementary material for: Perfluorooctanoate in Aqueous Urea Solutions: Micelle Formation, Structure, and Microenvironment
Source: Int J Mol Sci. 2019 Nov 16;20(22):5761. doi: 10.3390/ijms20225761 (PMC6888096; doi:10.3390/ijms20225761)
Supplement: Supplementary file 1 [file ijms-20-05761-s001.pdf]

Manuscript ID ijms-633013

Perfluorooctanoate in aqueous urea solutions: micelle formation, structure, and microenvironment

Samhitha Kancharla, Emmanuel Canales, Paschalis Alexandridis*
Chem&Biol Engineering, University at Buffalo, The State University of New York, Buffalo, NY 14260, USA

SUPPLEMENTARY INFORMATION

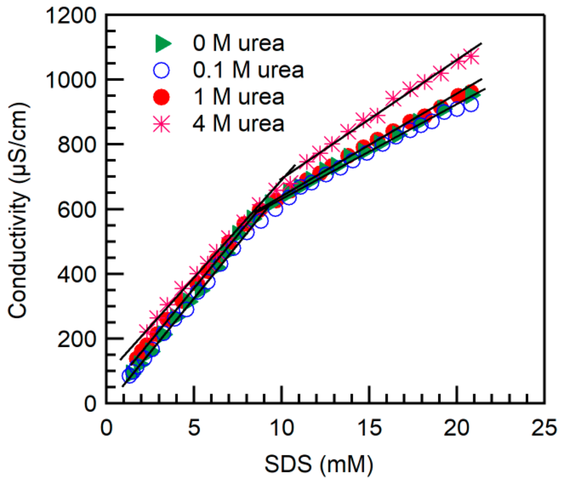

Figure S1. Conductivity of SDS aqueous solutions in the absence and in the presence of added urea as a function of surfactant concentration at 24 °C.

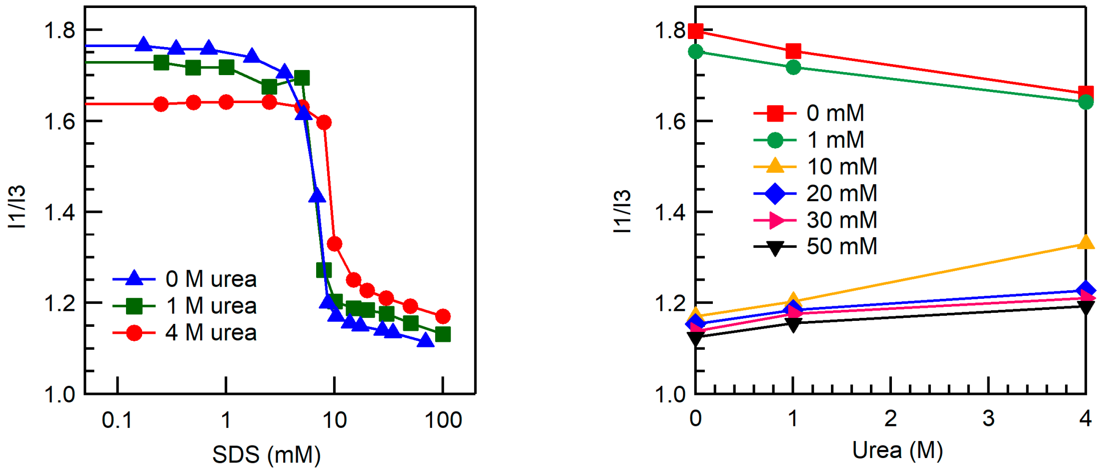

Figure S2. (left) Pyrene fluorescence intensity I1/I3 ratio of SDS aqueous solutions plotted versus surfactant concentration in the absence and in the presence of urea (at various urea concentrations shown inside the graph) (22 °C); (right) Variation with urea concentration of the pyrene fluorescence intensity I1/I3 ratio of SDS aqueous solutions at various SDS concentrations (shown inside the graph). The lines connecting the data points are meant as guides to the eye.

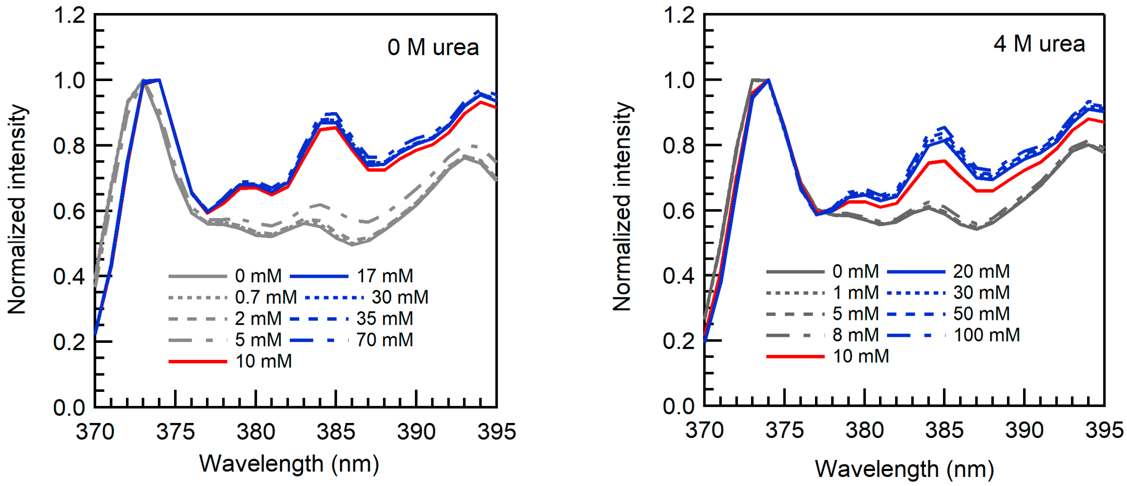

Figure S3. Normalized pyrene monomer emission spectra of SDS aqueous solutions in the absence (left) and in the presence of urea (right). The spectra have been normalized by dividing the intensity at each wavelength with the intensity of the first peak (I1). The grey lines (——, ·········, - - - - -, — - — -) indicate the spectra of pyrene at SDS concentrations below the CMC, the red line (——) is at SDS concentration near the CMC, and the blue lines (——, ·········, - - - - -, — - — -) are at SDS concentrations above the CMC.
